# Early diagnosis of solitary functioning kidney: comparing the prognosis of kidney agenesis and multicystic dysplastic kidney

**DOI:** 10.1007/s00467-024-06360-2

**Published:** 2024-04-15

**Authors:** Hana Flogelova, Katerina Bouchalova, Oldrich Smakal, Jan Halek, Katerina Langova, Katerina Cizkova

**Affiliations:** 1 Department of Pediatrics, Faculty of Medicine and Dentistry, Palacky University Olomouc and University Hospital Olomouc, Zdravotniku 248/7, 779 00 Olomouc, Czech Republic; 2 Department of Urology, University Hospital Olomouc, Olomouc, Czech Republic; 3 Department of Neonatology, University Hospital Olomouc, Olomouc, Czech Republic; 4 Department of Medical Biophysics, Faculty of Medicine and Dentistry, Palacky University Olomouc, Olomouc, Czech Republic; 5 Department of Histology and Embryology, Faculty of Medicine and Dentistry, Palacky University Olomouc, Olomouc, Czech Republic

**Keywords:** Functional solitary kidney, Unilateral kidney agenesis, Unilateral multicystic dysplastic kidney, Glomerular filtration rate, CAKUT

## Abstract

**Background:**

Individuals with congenital solitary functioning kidney (SFK) are at an increased risk of kidney damage. According to some studies, the risk is higher in unilateral kidney agenesis (UKA) than in unilateral multicystic dysplastic kidney (UMCDK). We hypothesized that with early detection of children with UKA and UMCDK, there would be no difference in the presence of hypertension, proteinuria, and reduced glomerular filtration rate (GFR) between UKA and UMCDK.

**Methods:**

Based on a long-term follow-up protocol, we evaluated a cohort of 160 children followed from birth for SFK (84 with UKA and 76 with UMCDK) detected by prenatal or routine neonatal ultrasound screening. Hypertension, proteinuria, and reduced GFR were monitored as markers of kidney damage. We compared the characteristics and outcomes of the subgroups of children with UKA and UMCDK.

**Results:**

GFR was reduced in 42 (26.2%) children, of whom 41 showed only mild reduction. Hypertension and proteinuria were found in 22 (13.8%) and 14 (8.8%) children, respectively. Combined kidney damage was present in 57 (35.6%) children. The UMCDK and UKA subgroups differed in GFR at final examination, with UMCDK patients being significantly more likely to have normal GFR compared to UKA patients (82% vs. 67%; *p* = 0.039).

**Conclusions:**

One third of the children showed signs of SFK damage, albeit mild. Patients with UKA had reduced GFR significantly more often than those with UMCDK, but did not differ in the rates of hyperfiltration injury or congenital anomalies of the kidneys and urinary tract (CAKUT) in SFK.

**Graphical Abstract:**

**Figure:**
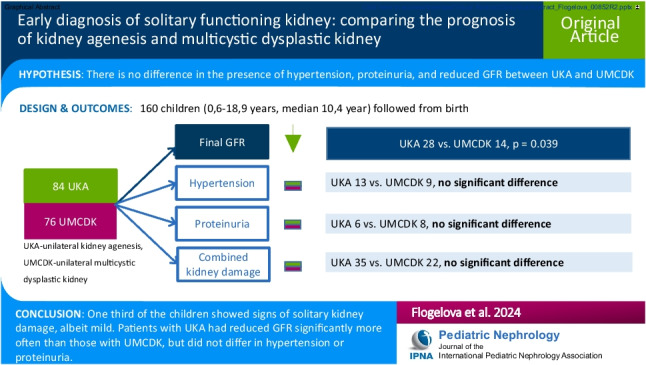
A higher resolution version of the Graphical abstract is available as Supplementary information

**Supplementary Information:**

The online version contains supplementary material available at 10.1007/s00467-024-06360-2.

## Introduction

In children, 30–50% of chronic renal failure cases are caused by congenital anomalies of the kidneys and urinary tract (CAKUT) [1, 2]. The risk of kidney damage is mainly present in children with bilateral kidney anomalies or solitary functioning kidney (SFK), especially if SFK is associated with CAKUT, such as high-grade vesicoureteral reflux (VUR) or obstructive defects. However, individuals with congenital SFK without associated CAKUT also have a higher risk of renal function decline over their lifetime than those with both kidneys. Published papers are inconsistent as to whether already in childhood this risk is significant [3] or low [4–6]. Similar discrepancies are seen in the presence of hyperfiltration injury to the solitary kidney (hypertension, proteinuria). One explanation for the different solitary kidney outcomes in published studies is the use of varied inclusion criteria. Diagnosing SFK during ultrasound (US) screening or only after clinical manifestations is likely to play a role.

This study aimed to determine the frequency of SFK damage in children followed from birth for unilateral kidney agenesis (UKA) or unilateral multicystic dysplastic kidney (UMCDK). We hypothesized that children with SFK in the cohort would have a good prognosis regarding kidney damage because of regular monitoring leading to early diagnosis. Another objective was to identify the characteristics that distinguish the UKA subgroup from the UMCDK subgroup and whether the frequency of SFK damage is different in these subgroups. Although a worse prognosis for UKA is reported in the literature [7, 8], we assumed that because of the early detection of children with asymptomatic UKA, there would be no difference in the presence of hypertension, proteinuria, and reduced glomerular filtration rate (GFR) between UKA and UMCDK patients. Based on the findings, we planned to optimize the follow-up protocol for children with SFK.

## Material and methods

### Patients

Using a long-term follow-up protocol, we retrospectively evaluated a cohort of patients followed at our center between 2000 and 2023 for a congenital SFK caused by UKA or UMCDK. Some of these patients (46 out of 160) had been enrolled in our earlier prospective multicenter study entitled “Renal Parenchymal Thickness in Children with Solitary Functioning Kidney” [9]. The current cohort of patients included children with UKA or UMCDK identified by prenatal or postnatal US screening. In the Czech Republic, prenatal US screening is performed as part of recommended gynecological procedures in all pregnant women and is covered by insurance. With regard to renal defects, screening at 20–22 and then at 30–32 weeks’ gestation is essential. In addition to regular prenatal US screening, US screening for renal defects is performed in all newborns aged 3 to 4 days in our center.

In infancy, SFK was confirmed by DMSA static renal scintigraphy and was defined as 100% unilateral function. In patients with significant urinary tract dilatation, MAG3 diuretic renal scintigraphy, as an alternative to DMSA scintigraphy, was performed to confirm SFK.

The exclusion criterion was having diseases other than CAKUT that could affect renal function in the neonatal period, such as sepsis or perinatal asphyxia. The cohort size was determined by a statistician based on literature data and a pilot analysis of some of our patient cohort.

### Indicators of kidney damage

The indicators of kidney damage were reduced GFR and signs of hyperfiltration injury, that is, hypertension and proteinuria. The category of combined kidney damage comprised children with any of the above indicators, that is, reduced GFR or hypertension or proteinuria.

The GFR was calculated using two formulas based on the child’s serum creatinine level, height, age, and sex, namely the Schwartz formula [10, 11] and CKiDU25 [12]. In children aged 2 years or older, GFR < 90 mL/min/1.73 m^2^ was considered reduced and GFR < 60 mL/min/1.73 m^2^ was considered moderately reduced. The reduced GFR category included children whose GFR was found to be reduced using both formulas. According to the KDIGO guidelines, long-term GFR < 90 mL/min/1.73 m^2^ is referred to as stage 2 chronic kidney disease (CKD 2) and GFR 60–89 mL/min/1.73 m^2^ as stage 3 chronic kidney disease (CKD 3). In children younger than 2 years of age, GFR was assessed according to the National Kidney Foundation recommendations [11].

Casual oscillometric blood pressure (BP) measurements were obtained in all children during regular outpatient visits. In patients under 3 years of age, only the oscillometric method with a proper cuff was used (noninvasive BP measurements using a vital sign monitor). In children older than 3 years of age, sitting oscillometric BP measurements were primarily taken following 3 to 5 min of resting. In case of high BP, measurements were repeated twice, taken in both arms, and the second and third readings were averaged. If the resulting value was still high, measurements were repeated in a similar manner using the auscultatory method. For children over 5 years of age with high casual BP, ambulatory BP monitoring (ABPM) was added.

Hypertension was defined as BP ≥ 95th percentile for the sex, age, and height of the child [13] at casual measurement and BP ≥ 95th percentile for the sex and height of the child at ABPM [14]. Children with hypertension at casual BP measurement had other secondary causes of hypertension excluded.

In addition, some patients older than 5 years (65 out of 160) underwent 24-h ABPM as a screening method for detecting masked or nocturnal hypertension.

Proteinuria was assessed by calculating the urine protein–creatinine ratio (uPCR) and/or urine albumin–creatinine ratio (uACR) in early morning urine samples. Proteinuria was defined as uPCR > 20 mg/mmol and/or uACR > 3 mg/mmol in children aged 2 years or older and as uPCR > 50 mg/mmol and/or uACR > 10 mg/mmol in children younger than 2 years [15].

Children with hypertension and repeated proteinuria or albuminuria were treated with ACE inhibitors or angiotensin receptor blockers. These children were categorized as having hypertension or proteinuria, even though their BP or urine proteins were controlled with medication.

### Risk factors

Following on from previous studies on congenital SFK, we investigated the following risk factors: sex, SFK side, immaturity (preterm birth before 37 weeks’ gestation), low birth weight (< 2500 g), GFR at initial examination, SFK length, CAKUT in SFK, urinary tract infection (UTI), body mass index (BMI) at final examination, hypertension, proteinuria/albuminuria, and urinary beta-2 microglobulin (U-B2M).

SFK length was measured by US and classified into percentile intervals according to the mean kidney length by age, as described by Akhavan et al. [16].

CAKUT in the solitary kidney was diagnosed by US and, in selected patients, by MAG3 diuretic renal scintigraphy to detect pelviureteric junction obstruction (PUJO) and by micturating cystourethrography (MCUG) to detect VUR. The severe CAKUT category included obstructive uropathy of SFK (PUJO, primary obstructive megaureter) requiring surgery or nonobstructive hydronephrosis with an anterior–posterior diameter ≥ 15 mm or megaureter ˃ 7 mm. Also, grade 4–5 VUR and refluxing megaureter were classified as severe CAKUT.

UTI was defined as the presence of pyuria and significant bacteriuria.

BMI was assessed using national reference charts for sex and age [17] and categorized as normal, overweight (> 85th percentile and ≤ 97th percentile), and obese (> 97th percentile).

The indicator of tubular injury was U-B2M, with levels above 0.202 mg/L being considered elevated by our laboratory.

### Follow-up protocol

The follow-up protocol was similar for all patients, but evolved over the years. Children who were diagnosed with UKA or UMCDK by prenatal or postnatal US screening were followed in our outpatient nephrology unit. In the case of normal postnatal US findings in the functional kidney, the first appointment was scheduled at approximately 3 months of age, which included a follow-up US examination and DMSA static renal scintigraphy to confirm the function of only one kidney. At the same time, GFR was measured (using serum creatinine levels) and urine tests were performed, including uPCR and/or uACR. Parents were informed about the symptoms of UTI. In selected children with dilatation of the SFK pelvicalyceal system, antibiotic prophylaxis for UTI was administered. With normal US findings in SFK, the follow-up US scan, urine tests, and BP measurements were scheduled at 1 year of age, then annually until 5 years of age, and every 2 years thereafter. Initially, over the first 7 years of following the cohort, MCUG was indicated in all children with SFK. Later, the follow-up protocol was changed so that MCUG was only performed in selected patients—those with SFK ureteral dilatation on US, SFK length below the 5th percentile, or UTI.

Initially, proteinuria was monitored, later albuminuria was added, and in the last 5 years, U-B2M was also measured. Preventive ABPM was performed only in the last 10 years. In the case of any pathological finding (SFK dilatation on US, proteinuria, reduced GFR, hypertension, UTI), check-ups were more frequent. Patients with obstructive SFK defects underwent early surgery by consensus of a nephrologist and a urologist. Surgery was also indicated for high-grade VUR and febrile UTI occurring despite antibiotic prophylaxis.

Patients were followed at our center until 18–19 years of age. After that, further follow-up was recommended, either by a general practitioner or, if kidney damage was suspected, by a specialist, usually an adult nephrologist. The detailed follow-up protocol is shown in Figure S1 in the Supplementary Material.

### Statistical analysis

Two independent samples were compared using Fisher’s exact test (qualitative data) and Mann–Whitney U-test (numerical and ordinal data).

Risk factors for reduced GFR and hypertension were assessed with odds ratio (OR) and 95% confidence interval (95% CI). The former was calculated by logistic regression. In addition, multivariate logistic regression was used to assess risk factors for reduced GFR and hypertension. In both cases, the model included the following variables: sex, BMI, immaturity, low birth weight, recurrent UTI, hypertension, proteinuria, elevated U-B2M, SFK side, SFK length at 1 year of age, GFR at initial examination, and severe CAKUT. The model was developed using the forward stepwise method (likelihood ratio).

All tests were performed at a significance level of 0.05. Statistical analyses were performed with IBM SPSS Statistics 23.0 (Armonk, NY: IBM Corp.).

## Results

**Fig. 1:**
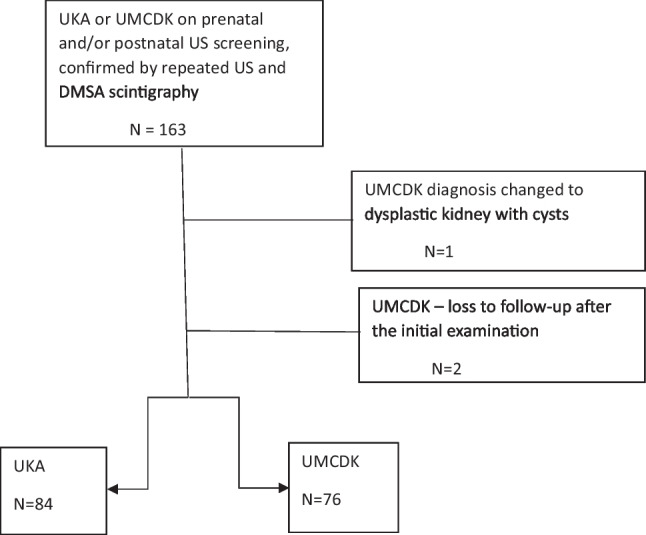
Identification of patients. Out of 163 children who were examined in an outpatient nephrology unit of a university hospital pediatric department based on pre and/or postnatal US screening and did not have neonatal sepsis or perinatal asphyxia, 160 were included in the study. The reasons for excluding three patients are stated. A total of 84 children had SFK due to UKA and 76 due to UMCDK

The inclusion criteria were met by 163 children with congenital SFK, of whom three were excluded (see Fig. 1). Their mean age at final examination was 10.4 years (median 10.1 years, SD 5.5 years). Detailed clinical data of the patients are shown in Table 1.

**Table 1:** Clinical characteristics of all children with congenital SFK at the beginning of the follow-up

*n* = 160	Count	%
Sex		
Male	101	63.1
Female	59	36.9
Immaturity	10	6.3
Low birth weight	12	7.5
Prenatal diagnosis	87	54.4
Etiology		
UMCDK	76	47.5
UKA	84	52.5
SFK side		
Right	84	52.5
Left	76	47.5
CAKUT in SFK	29	18.1
Severe CAKUT	11	6.9
CAKUT type		
Hydronephrosis	4	13.8
Megaureter	7	24.1
ADPKD	1	3.4
PUJO	2	6.9
Duplex kidney	1	3.4
VUR	14	48.3
Other anomalies	36	22.5
CNS	1	2.8
Female genitals	3	8.3
Male genitals	9	25.0
GIT	4	11.1
Cardiac	7	19.4
Cardiac, male genitals	1	2.8
Musculoskeletal	9	25.0
Musculoskeletal, male genitals	1	2.8
Musculoskeletal + mixed	1	2.8
US – SFK length, 3 months of age		
Below p50	9	6.2
p50–75	14	9.7
p75–95	64	44.1
Above p95	58	40.0
US – SFK length, 1 year of age		
Below p50	5	3.2
p50–75	13	8.3
p75–95	62	39.7
Above p95	76	48.7
GFR – initial		
Normal	151	94.4
Mildly reduced	9	5.6

*SFK* solitary functioning kidney, *UMCDK* unilateral multicystic dysplastic kidney, *UKA* unilateral kidney agenesis, *CAKUT* congenital anomalies of the kidneys and urinary tract, *ADPKD* autosomal dominant polycystic kidney disease, *PUJO* pelviureteric junction obstruction, *VUR* vesicoureteral reflux, *CNS* central nervous system, *GIT* gastrointestinal tract, *US* ultrasound, *GFR* glomerular filtration rate

As for the etiology of SFK, there were 76 children with UMCDK (47.5%) and 84 children with UKA (52.5%). Prenatally, SFK was detected in 54.4% of the 160 children; the others were diagnosed during postnatal US examination in the neonatal period.

CAKUT in the solitary kidney was seen in 18.1% of the 160 children, with VUR being the most common anomaly (48.3% of 25 children with CAKUT). MCUG was performed in 69 out of the 160 patients (43.1%). Eleven out of the 160 children (6.9%) had severe CAKUT, of whom nine were indicated for surgical management of the SFK defect. Five children underwent ureteral reimplantation for grade 4 VUR or refluxing megaureter, two were operated on for obstructive megaureter, and two underwent pyeloplasty for PUJO.

Anomalies of other organs were found in 22.5% of the 160 children. The most frequent were anomalies of the male genitals (two hypospadias, one seminal vesicle cyst, one cystic testicular dysplasia, four cryptorchidisms, and two Zinner syndromes) and the musculoskeletal system (three vertebral anomalies, one polydactyly, two cutaneous syndactylies, one radial club hand, one nail patella syndrome, and others). Cardiac anomalies included four atrial septal defects, three ventricular septal defects, and one pulmonary stenosis. Gastrointestinal tract (GIT) anomalies included two diaphragmatic hernias, one GIT duplication, and one esophageal atresia and anorectal atresia. Female genital anomalies included one Herlyn–Werner–Wunderlich syndrome (obstructed hemivagina and ipsilateral renal anomaly), one bicornuate uterus, and one double uterus and vaginal septum. One child with a central nervous system anomaly had agenesis of the corpus callosum and colpocephaly. For detailed information on anomalies of other organs, see Table S1 in the Supplementary Material.

Compensatory renal hypertrophy, monitored by measuring SFK length during US examination, was present in 40% of 145 children aged 3 months and 48.7% of 156 1-year-old children. Table S2 in the Supplementary Material shows that the risk factor for absent compensatory renal hypertrophy was anomaly of another organ (*p* = 0.03).

The initial GFR was reduced in 5.6% of the 160 children (minus 1 to 2 SDs from the mean) and normal in the rest of the cohort. No children had severely reduced GFR in the first months of life (less than minus 2 SDs).

### Kidney damage in the entire cohort

Table 2 shows the results for all children at the end of follow-up.

**Table 2:** Outcomes in all children with congenital SFK and comparison of the UMCDK and UKA subgroups

Variable	ALL		UMCDK		UKA		*p*
	*n* = 160	%	*n* = 76	%	*n* = 84	%	
Sex							
Male	101	63.1	48	63.2	53	63.1	1.000
Female	59	36.9	28	36.8	31	36.9	
SFK side							
Right	84	52.5	39	51.3	45	53.6	
Left	76	47.5	37	48.7	39	46.4	0.874
Immaturity	10	6.3	3	3.9	7	8.3	0.334
Low birth weight	12	7.5	5	6.6	7	8.3	0.769
Prenatal diagnosis	87	54.4	61	80.3	26	31	< 0.0001***
CAKUT in SFK	29	18.1	11	14.5	18	21.4	0.306
Severe CAKUT in SFK	11	6.9	5	6.6	6	7.1	1.000
Other anomalies	36	22.5	14	18.4	22	26.2	0.261
US – SFK length, 3 months of age							
Below p50	9	6.2	4	5.6	5	6.8	
p50–75	14	9.7	6	8.5	8	10.8	
p75–95	64	44.1	29	40.8	35	47.3	
Above p95	58	40.0	32	45.1	26	35.1	0.245
US – SFK length, 1 year of age							
Below p50	5	3.2	3	4.1	2	2.5	
p50–75	13	8.3	7	9.3	6	7.4	
p75–95	62	39.7	27	36	35	43.2	
Above p95	76	48.7	38	50.7	38	46.9	0.863
UTI	30	18.8	20	26.3	10	11.9	0.025*
Recurrent UTI	10	6.3	6	7.9	4	4.8	0.52
BMI							
Normal	117	73.1	58	76.3	59	70.2	
Overweight	29	18.1	13	17.1	16	19	0.350
Obesity	14	8.8	5	6.6	9	10.7	
Hypertension	22	13.8	9	11.8	13	15.5	0.647
Antihypertensives			12	15.8	9	10.7	0.360
Proteinuria/albuminuria	14	8.8	8	10.5	6	7.2	0.579
U-B2M elevation ****	18	11.3	7	11.7	11	17.5	0.448
GFR – initial							
Normal	151	94.4	71	93.4	80	95.2	
Mildly reduced	9	5.6	5	6.6	4	4.8	0.737
GFR – final							
Normal	118	73.8	62	81.6	56	66.7	
Mildly reduced	41	25.6	13	17.1	28	33.3	0.039*
Moderately reduced	1	0.6	1	1.3	0	0	
Combined kidney damage	57	35.6	22	28.9	35	41.7	0.101

*p< 0.05; *** *p* < 0.001; **** analysis only on 123 patients (60 UMCDK, 63 UKA)

*UMCDK* unilateral multicystic dysplastic kidney, *UKA* unilateral kidney agenesis, *SFK* solitary functioning kidney, *CAKUT* congenital anomalies of the kidneys and urinary tract, *US* ultrasound, *UTI* urinary tract infection, *BMI* body mass index, *U-B2M* urinary beta-2 microglobulin, *GFR* glomerular filtration rate

Renal function decline: Although GFR was reduced in a total of 42 out of the 160 children (26.2%), the reduction was mild (CKD 2) in 41 of them and moderate (CKD 3) in only one child (0.6%), a patient with UMCDK.

Hyperfiltration injury: Hypertension was observed in 22 out of the 160 children (13.8%) and proteinuria in 14 out of the 160 children (8.8%).

Combined kidney damage was present in 57 children (35.6%).

### Other indicators

Elevated BMI was found in 26.9% of the 160 children, with 18.1% being overweight and 8.8% being obese.

UTI developed in 18.8% of the 160 children, of whom 6.3% had recurrent UTI.

Some patients in the cohort (76.9%) also had U-B2M assessed. The indicator of tubular injury was elevated in 11.3% of 123 children; patients with severe CAKUT had significantly more often elevated U-B2M than the others (*p* < 0.01).

Compensatory renal hypertrophy was present in 61.4% of the 160 children at the end of follow-up.

In patients with UMCDK, involution of the multicystic dysplastic kidney was monitored by US examination and confirmed in 79.4% of 71 children with a mean age of 3.45 years.

### Comparison of the UMCDK and UKA subgroups

The numbers of children in the two subgroups were comparable (Table 1), as was the mean age, which was 10.3 years (SD 5.5) for UMCDK and 10.5 years (SD 5.4) for UKA (*p* = 0.78). Table 2 shows a statistical comparison of the clinical characteristics of the two subgroups. The UMCDK and UKA subgroups differed in three characteristics: GFR at final examination, prenatal diagnosis, and the presence of UTI. Patients with UMCDK were statistically significantly more likely to have normal GFR values than those with UKA (82% vs. 67%, *p* = 0.04). Children with UMCDK were statistically significantly more likely to have their renal defects detected prenatally (80% vs. 31%, *p* < 0.01). UTI was statistically significantly more frequent in patients with UMCDK than in those with UKA (26% vs. 12%, *p* = 0.03). No other significant differences were found. There was no difference in hyperfiltration injury (hypertension, proteinuria) or combined kidney damage. Finally, there was no significant difference in the presence of CAKUT.

### Risk factors for kidney damage

The risk factors for reduced GFR identified by logistic regression are shown in Table S3. A statistically significant protective factor was kidney length on US at 3 months and 1 year of age. Compensatory hypertrophy (kidney length above the 95th percentile) at 3 months of age and at 1 year of age reduced the odds ratio for reduced GFR to one third (0.356 and 0.330, respectively). Statistically significant risk factors for reduced GFR in the entire cohort were CAKUT (OR = 2.86), severe CAKUT (OR = 9.02), hypertension (OR = 4.32), proteinuria (OR = 6.11), need for antihypertensive/antiproteinuric medication (OR = 3.83), and reduced GFR at initial examination at the beginning of the follow-up (OR = 6.39).

Subsequently, the risk factors for reduced GFR were then evaluated using multivariate logistic regression. The results are shown in Table 3. The final model comprised two significant risk factors and one significant protective factor. The former were hypertension (OR = 9.34) and proteinuria (OR = 15.11). The protective factor was kidney length at 1 year greater than the 95th percentile (OR = 0.11).

**Table 3:** Risk (protective) factors for reduced GFR and hypertension–multivariate logistic regression

Independent variable: reduced GFR			
Dependent variables	OR	(95% CI)	*p*
Proteinuria/albuminuria	15.113	(2.429; 94.022)	0.004**
Hypertension	9.335	(2.106; 41.385)	0.003**
US – SFK length, 1 year of age	0.112	(0.032; 0.391)	0.001**
Independent variable: hypertension			
Dependent variables	OR	(95% CI)	*p*
BMI (normal vs. overweight, obesity)	5.487	(1.525; 19.743)	0.009**
GFR – initial	13.815	(1.468; 130.040)	0.022*

*p< 0.05; ** *p* < 0.01

*GFR* glomerular filtration rate, *OR* odds ratio, *CI* confidence interval, *US* ultrasound, *SFK* solitary functioning kidney, *BMI* body mass index

### Risk factors for hypertension

Table S4 shows the risk factors for hypertension. GFR at initial examination (OR = 16.88) and severe CAKUT (OR = 4.16) were found to be statistically significant risk factors. Table 3 shows the risk factors identified by multivariate logistic regression. In the final model, there were two significant factors, namely BMI (OR = 5.49) and GFR at initial examination (OR = 13.82), both of which are risk factors. Patient overweight or obesity increases the odds of developing hypertension, as does reduced GFR at initial examination.

## Discussion

Our cohort of children with SFK is specific in that they were diagnosed in the neonatal period based on both prenatal and routine postnatal US screening. Although the quality of prenatal US screening continues to improve, certain cases are still missed in the prenatal period. Children with asymptomatic UKA, who would likely improve the prognosis of patients with SFK, may go undetected for a long time and are not included in the populations studied.

The present study evaluated the presence of SFK damage in children regularly visiting a single nephrology center and compared the outcome of UKA and UMCDK. Combined prenatal and neonatal US screening allows for the earliest possible diagnosis of SFK, early follow-up, and early treatment of both CAKUT in SFK and proteinuria and hypertension. To the best of our knowledge, only one study has been published on congenital SFK diagnosed by combined prenatal and postnatal US screening, which did not compare the prognosis of UKA and UMCDK [18].

The main finding was that one third of the cohort showed signs of SFK damage, albeit mild, even though CAKUT in SFK was relatively rare. When comparing UKA and UMCDK, there was a difference in the frequency of reduced GFR, but no difference in any other characteristic.

Impaired SFK function, defined by GFR below 90 mL/min/1.73 m^2^ in the study, was found in a not negligible proportion (26.2%) of the entire cohort (mean follow-up of 10 years). On the other hand, only one child (0.6%) had moderately reduced GFR (CKD3); the others showed only mild GFR reduction (CKD2). When comparing the results with those of recent studies, the frequency of reduced GFR in our cohort is comparable to a study by Marzuillo et al. who reported reduced GFR in 21.4% of children [6]. Patients in our cohort have better GFR than those in the SOFIA study, in which 28% of children had CKD2, 3% had CKD3 or worse, and 1% required kidney transplantation [8]. By contrast, in a systematic review by Hutchinson et al., where GFR < 90 mL/min/1.73 m^2^ indicated pathological kidney function, only 8.4% of children had “worsened renal function.” However, it is not specified how the authors assessed “worsened renal function” in the studies included in the review [4]. We chose to consider GFR < 90 mL/min/1.73 m^2^ as impaired kidney function in our study as we believe that even individuals with mildly reduced GFR deserve long-term monitoring by a nephrologist. Since we are aware that the Schwartz formula for calculating GFR may underestimate GFR in some adolescent patients, GFR was also assessed using the CKiDU25 equation, and only individuals with both GFR values < 90 mL/min/1.73 m^2^ were categorized as having reduced GFR.

The frequency of hyperfiltration injury in the entire cohort was similar to that in the review by Hutchinson et al. and earlier studies [19]. In the present study, 13.8% of children had hypertension and 8.8% had proteinuria. The observed frequency of hypertension was higher due to the fact that 40.6% of the children in our cohort had their BP assessed by ABPM in addition to casual measurements. Of the 22 hypertensive patients, five had only nocturnal hypertension, which is impossible to detect without ABPM. Although according to the Italian Society of Pediatric Nephrology consensus recommendations, ABPM should not be used preventively in all children with SFK [19], we intend to continue preventive ABPM for the time being based on our results.

One of the main objectives of the present study was to compare the outcome of children with UKA and UMCDK. Some authors reported a worse outcome for UKA than for UMCDK [7, 8]. We hypothesized that this may be due to selection bias and that with our approach of diagnosing SFK by both prenatal and routine postnatal US, providing us with approximately equal numbers of UKA and UMCDK cases in the cohort, the prognosis of UKA would not be worse. However, this assumption was not confirmed. Although there was no statistically significant difference in the incidence of hypertension or proteinuria between UKA and UMCDK patients, we did demonstrate a difference in GFR at final examination. Individuals with UKA were statistically significantly more likely to have mildly reduced GFR, which was surprising given the absence of other differences. Both Matsell et al. and Groen in’t Woud et al. report CAKUT in SFK as a reason for the higher risk of kidney damage in UKA compared to UMCDK. In the present study, children with UKA had more frequent CAKUT in SFK than those with UMCDK, but this difference was not statistically significant. UTI was even more frequent in patients with UMCDK. The explanation for the slightly worse kidney function in children with UKA could be different embryonic development. Kidney agenesis manifests itself in earlier stages of development, while defects occurring later tend to be less severe. UKA results from the failure of the ureteric duct to contact and/or induce the metanephric mesoderm [20]. Studies involving conditional gene targeting in mice have pinpointed specific genes critical for normal kidney development, which have homologous counterparts linked to variants in humans, such as *SALL1*, *EYA1*, *PAX2*, or *RET* [21], particularly those associated with UKA. The impact of such gene variants can be widespread, affecting the contralateral kidney and leading to extrarenal anomalies. In a rat model of UKA, the contralateral kidney undergoes hypertrophy, but there is a reduced number of nephrons with hypertrophic glomeruli [22]. This may potentially account for the lower GFR observed in our subgroup of UKA patients. The appearance of fetal multicystic dysplastic kidneys suggests a disruption in established kidney induction after it has already initiated, affecting normal branching morphogenesis [23]. This scenario would more plausibly explain the development of a contralateral kidney, and there is a greater likelihood that it will be normal. Thus, the pathogenesis of these two conditions appears to be distinct, implying potential differences in outcomes.

While other authors reported CAKUT in 23.2 to 46% of SFK cases [3, 5–8, 18], the proportion in the present study was relatively low at 18.1%. This may be explained by the large number of children with asymptomatic SFK in our cohort. The most common CAKUT in SFK tends to be VUR, which needs to be diagnosed by MCUG. Initially, this examination was routinely performed in all children; later, it was limited to selected patients (SFK length below the 5th percentile, urinary tract dilatation, UTI) and thus may not have detected all SFK cases with VUR. However, our practice of performing MCUG is in line with recent recommendations [19].

Most publications report CAKUT as a risk factor for reduced GFR in solitary kidneys, whether UKA or UMCDK. In our entire cohort, CAKUT was also a risk factor, but hypertension and proteinuria were statistically more significant risk factors. The fact that CAKUT was diagnosed early in our patients and severe CAKUT was surgically treated in time may have mitigated the power of this risk factor. Surgery was mainly indicated for obstructive SFK defects. Our approach to the treatment of high-grade VUR to SFK evolved over time. In the first years of regular monitoring of children with SFK, one patient with grade 4 VUR, despite having an asymptomatic course, was indicated for ureteral reimplantation while still in infancy in an effort to prevent complicating UTI and to preserve kidney function as much as possible. Later, grade 4 VUR was surgically managed in children suffering from acute pyelonephritis despite antibiotic prophylaxis. In cases of low- or moderate-grade VUR, we opted for a conservative approach. According to a recent study by Marzuillo et al., high-grade VUR is more likely to resolve spontaneously in children with SFK than those with both kidneys, which speaks in favor of conservative treatment of VUR in patients with SFK [24].

An unpleasant finding was that 26.9% of the children in our cohort were overweight or even obese. Increased BMI was a statistically significant risk factor for hypertension; in some of the children with SFK, hypertension may have been caused by high BMI and not by hyperfiltration. Obesity itself, however, was not a risk factor for reduced GFR in the cohort, probably because not all children with increased BMI had hypertension. In patients with normal BMI, hypertension was likely due to hyperfiltration as other causes were ruled out. In any case, hypertension, whether due to renal hyperfiltration or obesity, was a significant risk factor for reduced GFR. Pediatricians and pediatric nephrologists should pay attention to the prevention of obesity in children with SFK, as it is a modifiable risk factor.

We do not expect neonatal US screening of CAKUT to be extended to all newborns to improve UKA detection. In our country, such screening is optional, and only 63% of all neonatal units perform it on their own initiative. The emphasis must be on the best possible prenatal US screening. Even children with normal US findings in SFK benefit from early referral to a pediatric nephrologist; the first screening should be performed between 2 and 3 months of age and should include US measurement of SFK length. Compensatory hypertrophy of SFK at 3 months and 1 year of age was a protective factor for reduced GFR in our cohort, consistent with observations by other authors [25, 26]. Currently, it is not considered necessary to confirm the function of only one kidney by DMSA static renal scintigraphy when the US findings are typical of UKA or UMCDK. Therefore, the follow-up protocol may be modified for children with or without compensatory hypertrophy of SFK. Patients with normal US findings in SFK, including compensatory hypertrophy, do not have to undergo DMSA static renal scintigraphy and collection of blood for creatinine tests in infancy. However, in addition to US, the initial nephrologic evaluation should include urine tests and educating parents about the symptoms of UTI to allow for early diagnosis and treatment. The frequency of US scans of SFK established in the study has proven beneficial and will be continued, as will antibiotic prophylaxis for UTI in selected patients with CAKUT in SFK and close collaboration with a pediatric urologist to indicate early surgical management of obstructive SFK defects. The children in the cohort continue to undergo preventive ABPM as part of a prospective study. We hypothesize that in our patients, GFR was favorably influenced by early treatment of hypertension and proteinuria.

The present study has several limitations, one of them being its retrospective design. Even though a follow-up protocol was used, the final data analysis was retrospective. Another limitation is that some patients (13 out of 42) were classified as having reduced GFR based on their last estimated GFR without repeated measurements to confirm that. Similarly, proteinuria was not confirmed by repeated measurements in four out of 14 children. This could have overestimated the prevalence of patients with kidney damage.

A certain limitation of the study is that when comparing the UKA and UMCDK phenotypes, it cannot be ruled out that UMCDK involution may occur before gestational week 20 when UMCDK is usually diagnosed. It is therefore possible that some of the UKA cases in the cohort were originally UMCDK and were thus misclassified, distorting the comparison of both SFK phenotypes. The literature provides data on UMCDK involution during pregnancy, albeit not during its first half [27].

In conclusion, this study has contributed to knowledge about congenital SFK by finding that even in a cohort followed from birth, with a large proportion of asymptomatic SFK cases, one third of the children showed signs of SFK damage. In most of them, however, the damage was mild. Patients with UKA had significantly more often reduced GFR compared to those with UMCDK, but did not differ in hyperfiltration injury or the frequency of CAKUT in SFK.

### Supplementary Information

Below is the link to the electronic supplementary material.

Graphical Abstract (PPTX 79 KB)

Supplementary file2 *body height and weight measured on each visit to the outpatient unit. **genetic tests in selected anomalies of other organs (musculoskeletal, gastrointestinal etc.). SFK - solitary functioning kidney, UKA - unilateral kidney agenesis, UMCDK -unilateral multicystic dysplastic kidney, US - ultrasound, BP - blood pressure, MCUG - micturating cystourethrography, eGFR - estimated glomerular filtration rate, PCR - protein/creatinine ratio, ACR - albumin/creatinine ratio, PUJO - pelviureteric junction obstruction (DOCX 62 KB)

Supplementary file3 (DOCX 18 KB)

Supplementary file4 (DOCX 15 KB)

Supplementary file5 (DOCX 18 KB)

Supplementary file6 (DOCX 18 KB)

## Data Availability

The datasets generated and analyzed during the current study are available from the corresponding author upon reasonable request.
