# Supplementary material for: Early diagnosis of solitary functioning kidney: comparing the prognosis of kidney agenesis and multicystic dysplastic kidney
Source: Pediatr Nephrol. 2024 Apr 15;39(9):2645–54. doi: 10.1007/s00467-024-06360-2 (PMC11272688; doi:10.1007/s00467-024-06360-2)
Supplement: Supplementary file 1 — Graphical Abstract (PPTX 79 KB) [file 467_2024_6360_MOESM1_ESM.pptx]

## Slide 1
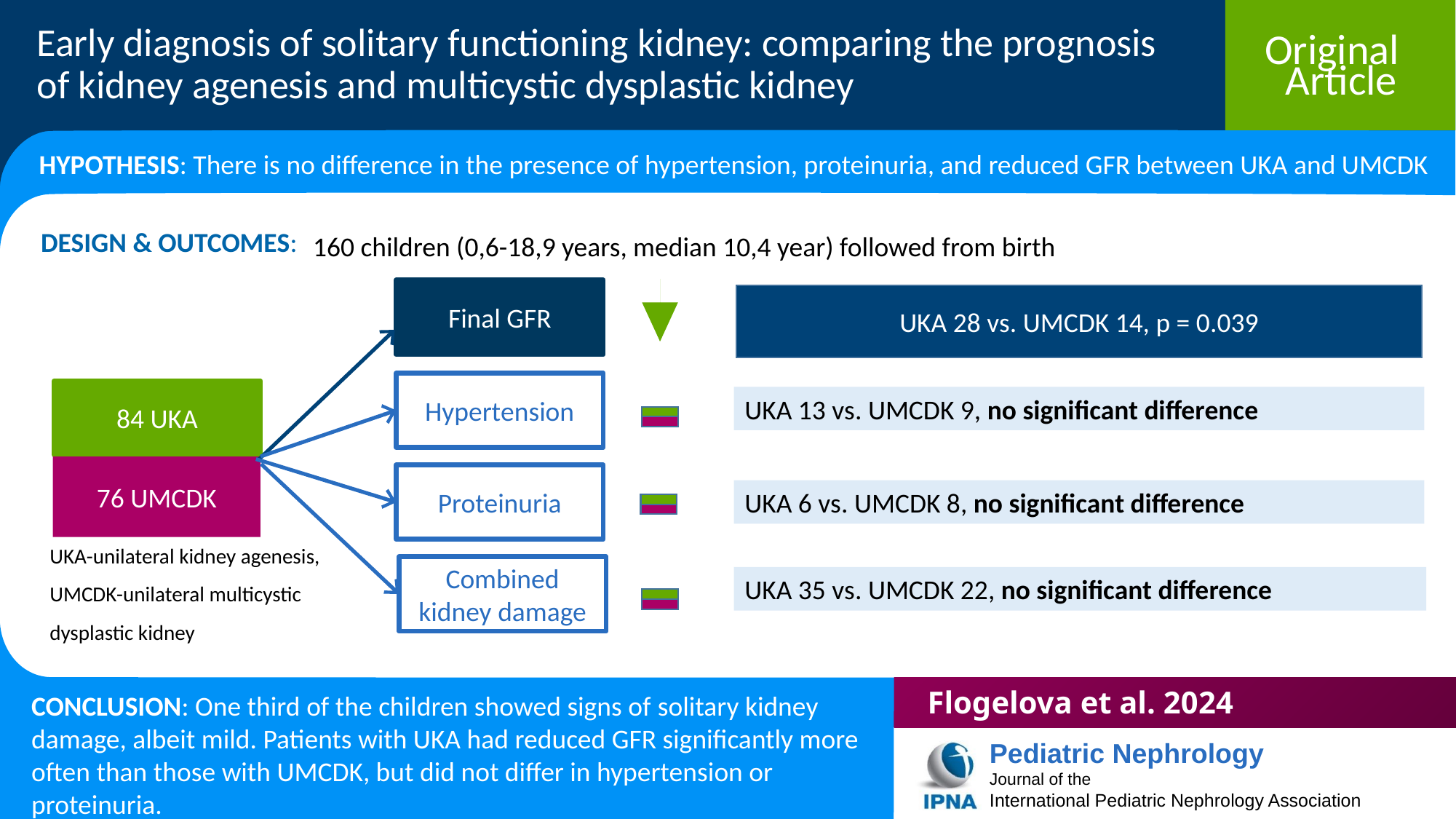

Early diagnosis of solitary functioning kidney: comparing the prognosis
of kidney agenesis and multicystic dysplastic kidney
HYPOTHESIS: There is no difference in the presence of hypertension, proteinuria, and reduced GFR between UKA and UMCDK
160 children (0,6-18,9 years, median 10,4 year) followed from birth
DESIGN & OUTCOMES:
Final GFR
UKA 28 vs. UMCDK 14, p = 0.039
Hypertension
84 UKA
UKA 13 vs. UMCDK 9, no significant difference
76 UMCDK
Proteinuria
UKA 6 vs. UMCDK 8, no significant difference
UKA-unilateral kidney agenesis,
UMCDK-unilateral multicystic
dysplastic kidney
Combined kidney damage
UKA 35 vs. UMCDK 22, no significant difference
Flogelova et al. 2024
CONCLUSION: One third of the children showed signs of solitary kidney damage, albeit mild. Patients with UKA had reduced GFR significantly more often than those with UMCDK, but did not differ in hypertension or proteinuria.
