# Supplementary material for: Early diagnosis of solitary functioning kidney: comparing the prognosis of kidney agenesis and multicystic dysplastic kidney
Source: Pediatr Nephrol. 2024 Apr 15;39(9):2645–54. doi: 10.1007/s00467-024-06360-2 (PMC11272688; doi:10.1007/s00467-024-06360-2)
Supplement: Supplementary file 2 — Supplementary file2 *body height and weight measured on each visit to the outpatient unit. **genetic tests in selected anomalies of other organs (musculoskeletal, gastrointestinal etc.). SFK - solitary functioning kidney, UKA - unilateral kidney agenesis, UMCDK -unilateral multicystic dysplastic kidney, US - ultrasound, BP - blood pressure, MCUG - micturating cystourethrography, eGFR - estimated glomerular filtration rate, PCR - protein/creatinine ratio, ACR - albumin/creatinine ratio, PUJO - pelviureteric junction obstruction (DOCX 62 KB) [file 467_2024_6360_MOESM2_ESM.docx]

**Fig. S1 Follow-up protocol for children with SFK**

UKA/UMCDK detected by pre- and/or postnatal US (all newborns aged 3 or 4 days)

Functioning cystic kidney - hydronephrosis or PUJO, not UMCDK

Contralateral functioning kidney only

From 4 weeks of age: MAG3 diuretic scintigraphy to determine relative renal function

UMCDK

US every 6 months in the 1st year of age, then once a year until 5 years of age, then once every 2 years. BP. Urine: chemistry+sediment, PCR, ACR. Once every 2-3 years eGFR. *

Repeated US by a pediatric radiologist, individual management (MCUG, MAG3 diuretic scintigraphy)

US: UMCDK cannot be distinguished from hydronephrosis

Girls: at 10-11 years gynecologic examination to rule out genital tract anomaly

US at around 1 year of age, then once a year until 5 years of age, then once every 2 years. BP. Urine: chemistry+sediment, PCR, ACR. Once every 2-3 years eGFR. *

2-6 months of age: * multicystic kidney size, US -contralateral kidney length, echogenicity and parenchymal thickness, DMSA scinfigraphy, eGFR, urine: chemistry+sediment, PCR, ACR, **

From 18-19 years of age

- damaged SFK – treated by adult nephrologist

- normal SFK – visits to general practicioner

2-6 months of age: * US – kidney length, echogenicity and parenchymal thickness, DMSA scinfigraphy, eGFR, urine: chemistry+sediment, PCR, ACR, **

US: UMCDK, normal finding in contralateral SFK

Dilatation of the SFK pelvicalyceal system or urinary bladder pathology

UKA

Normal US finding in SFK
