# Supplementary material for: Early diagnosis of solitary functioning kidney: comparing the prognosis of kidney agenesis and multicystic dysplastic kidney
Source: Pediatr Nephrol. 2024 Apr 15;39(9):2645–54. doi: 10.1007/s00467-024-06360-2 (PMC11272688; doi:10.1007/s00467-024-06360-2)
Supplement: Supplementary file 3 — Supplementary file3 (DOCX 18 KB) [file 467_2024_6360_MOESM3_ESM.docx]

| Table S1 Anomalies of other organs in children with SFK | |  |  |  |
| --- | --- | --- | --- | --- |
|  |  |  |  |  |
|  | all children |  | UKA | UMCDK |
|  | n=160 |  | n=84 | n=76 |
| Anomalies | count | % | count | count |
|  | 36 | 22.5 | 22 | 14 |
| CNS | 1 | 2.8 |  |  |
| agenesis of the corpus callosum, colpocephaly |  |  |  | 1 |
| female genitals | 3 | 8.3 |  |  |
| Herlyn-Werner-Wunderlich syndrome* |  |  | 1 |  |
| bicornuate uterus |  |  | 1 |  |
| double uterus and vaginal septum |  |  |  | 1 |
| male genitals | 9 | 25.0 |  |  |
| hypospadias |  |  | 1 | 1 |
| seminal vesicle cysts |  |  |  | 1 |
| cystic testicular dysplasia |  |  |  | 1 |
| Zinner syndrome |  |  | 1 |  |
| cryptorchidism |  |  | 2 | 2 |
| GIT | 4 | 11.1 |  |  |
| diaphragmatic hernia |  |  | 2 |  |
| GIT duplication, pyloric stenosis |  |  |  | 1 |
| esophageal atresia, anorectal atresia |  |  | 1 |  |
| cardiac | 7 | 19.4 |  |  |
| atrial septal defect |  |  | 2 | 2 |
| ventricular septal defect |  |  | 2 | 1 |
| cardiac + male genitals | 1 | 2.8 |  |  |
| pulmonary stenosis, cryptorchidism |  |  | 1 |  |
| musculoskeletal | 9 | 25.0 |  |  |
| vertebral anomalies |  |  | 3 |  |
| cutaneous syndactyly |  |  | 1 | 1 |
| radial club hand |  |  | 1 |  |
| nail patella syndrome |  |  |  | 1 |
| hip dysplasia |  |  |  | 1 |
| congenital clubfoot |  |  | 1 |  |
| musculoskeletal + male genitals | 1 | 2.8 |  |  |
| polydactyly, Zinner syndrome |  |  | 1 |  |
| musculoskeletal + mixed | 1 | 2.8 |  |  |
| joint hypermobility, hypoacusis, myopia** |  |  | 1 |  |
|  |  |  |  |  |
| *also known as OHVIRA syndrome (double uterus, vaginal septum, hematocolpos, hematometra,  hematosalpinx) | | | | |
| **not genetically confirmed either Ehlers Danlos syndrome or Stickler syndrome | | | |  |
