# Supplementary material for: Early diagnosis of solitary functioning kidney: comparing the prognosis of kidney agenesis and multicystic dysplastic kidney
Source: Pediatr Nephrol. 2024 Apr 15;39(9):2645–54. doi: 10.1007/s00467-024-06360-2 (PMC11272688; doi:10.1007/s00467-024-06360-2)
Supplement: Supplementary file 4 — Supplementary file4 (DOCX 15 KB) [file 467_2024_6360_MOESM4_ESM.docx]

| Table S2 Relationship between compensatory hypertrophy of SFK and CAKUT in SFK and anomalies of other organs | | | |
| --- | --- | --- | --- |
|  |  |  |  |
|  | Compensatory hypertrophy of SFK | | |
| UKA and UMCDK together | not present | present | p |
| number of children, n=158 | 60 | 98 |  |
| all with CAKUT | 14 (23.3) | 16 (16.3) | 0.301 |
| all other anomalies | 20 (33.3) | 17 (17.3) | 0.032* |
| CAKUT and/or other anomalies | 27 (45.0) | 29 (29.6) | 0.06 |
| without CAKUT or other anomalies | 33 (55.0) | 69 (70.4) | 0.06 |
| *p< 0.05 |  |  |  |
| Percentages stated in brackets. | |  |  |
|  |  |  |  |
| Compensatory hypertrophy was classified as present if detected by US at 3 months and/or 1 year of age.  The distribution of frequencies for the two patient groups was determined by Fisher’s exact test.  The difference was considered statistically significant if the p-value was below 0.05 (marked with an asterisk). | | | |
