# Supplementary material for: Early diagnosis of solitary functioning kidney: comparing the prognosis of kidney agenesis and multicystic dysplastic kidney
Source: Pediatr Nephrol. 2024 Apr 15;39(9):2645–54. doi: 10.1007/s00467-024-06360-2 (PMC11272688; doi:10.1007/s00467-024-06360-2)
Supplement: Supplementary file 5 — Supplementary file5 (DOCX 18 KB) [file 467_2024_6360_MOESM5_ESM.docx]

Table S3 Risk (protective) factors for reduced GFR

|  |  | GFR- final |  |  |  |  |  |
| --- | --- | --- | --- | --- | --- | --- | --- |
|  | reduced |  | normal |  | OR | (95% CI) | p |
|  | Count | % | Count | % |  |  |  |
| Sex - male/female | 28 / 14 | 66.7 / 33.3 | 73 / 45 | 61.9 / 38.1 | 1.233 | (0.587; 2.587) | 0.710 |
| BMI |  |  |  |  |  |  |  |
| normal | 28 | 66.7 | 89 | 75.4 |  |  |  |
| overweight, obesity | 14 | 33.3 | 29 | 24.6 | 0.652 | (0.303; 1.402) | 0.312 |
| Immaturity | 3 | 7.1 | 7 | 5.9 | 1.220 | (0.301; 4.951 | 0.723 |
| Low birth weight | 3 | 7.1 | 9 | 7.6 | 0.932 | (0.240; 3.618) | 1.000 |
| CAKUT in SFK | 13 | 31 | 16 | 13.6 | 2.858 | (1.234; 6.620) | 0.019* |
| Severe CAKUT in SFK | 8 | 19 | 3 | 2.5 | 9.020 | (2.267; 35.887) | 0.001** |
| UTI | 8 | 19 | 22 | 18.6 | 1.027 | (0.418; 2.522) | 1.000 |
| Recurrent UTI | 4 | 9.5 | 6 | 5.1 | 1.965 | (0.526; 7.338) | 0.292 |
| Hypertension | 12 | 28.6 | 10 | 8.5 | 4.320 | (1.702; 10.967) | 0.003** |
| Proteinuria/albuminuria | 9 | 21.4 | 5 | 4.3 | 6.109 | (1.915; 19.490) | 0.002** |
| U-B2M elevation**** | 6 | 20.7 | 12 | 12.8 | 1.783 | (0.603; 5.268) | 0.367 |
| SFK side |  |  |  |  |  |  |  |
| right | 23 | 54.8 | 61 | 51.7 |  |  |  |
| left | 19 | 45.2 | 57 | 48.3 | 1.131 | (0.558; 2.293) | 0.857 |
| US - SFK length,  3 months of age |  |  |  |  |  |  |  |
| ≤ p95 | 27 | 77.1 | 60 | 54.5 |  |  |  |
| > p95 | 8 | 22.9 | 50 | 45.5 | 0.356 | (0.148; 0.852) | 0.018* |
| US - SFK length,  1 year of age |  |  |  |  |  |  |  |
| ≤ p95 | 29 | 70.7 | 51 | 44.3 |  |  |  |
| > p95 | 12 | 29.3 | 64 | 55.7 | 0.330 | (0.153; 0.710) | 0.003** |
| Antihypertensives | 11 | 26.2 | 10 | 8.5 | 3.832 | (1.490; 9.859) | 0.007** |
| GFR - initial |  |  |  |  |  |  |  |
| reduced | 6 | 14.3 | 3 | 2.5 |  |  |  |
| normal | 36 | 85.7 | 115 | 97.5 | 6.389 | (1.520; 26.846) | 0.011* |

*p<0.05; **p<0.01; **** analysis only on 123 patients (60 UMCDK, 63 UKA)
