# Supplementary material for: Early diagnosis of solitary functioning kidney: comparing the prognosis of kidney agenesis and multicystic dysplastic kidney
Source: Pediatr Nephrol. 2024 Apr 15;39(9):2645–54. doi: 10.1007/s00467-024-06360-2 (PMC11272688; doi:10.1007/s00467-024-06360-2)
Supplement: Supplementary file 6 — Supplementary file6 (DOCX 18 KB) [file 467_2024_6360_MOESM6_ESM.docx]

Table S4 Risk (protective) factors for hypertension

|  |  | Hypertension | |  |  |  |  |
| --- | --- | --- | --- | --- | --- | --- | --- |
|  | yes |  | no |  | OR | (95% CI) | p |
|  | Count | % | Count | % |  |  |  |
| Sex -male/female | 16 / 6 | 72.7 / 27.3 | 85/53 | 61.6 / 38.4 | 1.663 | (0.612; 4.515) | 0.352 |
| BMI |  |  |  |  |  |  |  |
| normal | 13 | 59.1 | 104 | 75.4 |  |  |  |
| overweight, obesity | 9 | 40.9 | 34 | 24.6 | 0.472 | (0.186; 1.302) | 0.124 |
| Immaturity | 1 | 4.5 | 9 | 6.5 | 0.683 | (0.082; 5.668) | 1.000 |
| Low birth weight | 1 | 4.5 | 11 | 8.0 | 0.550 | (0.067; 4.483) | 1.000 |
| CAKUT in SFK | 7 | 31.8 | 22 | 15.9 | 2.461 | (0.900; 6.731) | 0.081 |
| Severe CAKUT in SFK | 4 | 18.2 | 7 | 5.1 | 4.159 | (1.107; 15.624) | 0.047* |
| UTI | 3 | 13.6 | 27 | 19.6 | 0.649 | (0.179; 2.354) | 0.769 |
| Recurrent UTI | 2 | 9.1 | 8 | 5.8 | 1.625 | (0.322; 8.207) | 0.629 |
| Proteinuria/albuminuria | 3 | 13.6 | 11 | 8.0 | 1.809 | (0.462; 7.080) | 0.414 |
| U-B2M elevation**** | 3 | 23.1 | 15 | 13.6 | 1.900 | (0.468; 7.708) | 0.404 |
| SFK side |  |  |  |  |  |  |  |
| right | 13 | 59.1 | 71 | 51.4 |  |  |  |
| left | 9 | 40.9 | 67 | 48.6 | 1.363 | (0.547; 3.397) | 0.647 |
| US - SFK length, 3 months of age |  |  |  |  |  |  |  |
| ≤ p95 | 13 | 68.4 | 74 | 58.7 |  |  |  |
| > p95 | 6 | 31.6 | 52 | 41.2 | 1.523 | (0.543; 4.266) | 0.464 |
| US - SFK length, 1 year of age |  |  |  |  |  |  |  |
| ≤ p95 | 11 | 50.0 | 69 | 51.5 |  |  |  |
| > p95 | 11 | 50.0 | 65 | 48.5 | 1.062 | (0.431; 2.616) | 1.000 |
| GFR - initial |  |  |  |  |  |  |  |
| mildly reduced | 6 | 27.3 | 3 | 2.2 |  |  |  |
| normal | 16 | 72.7 | 135 | 97.8 | 16.880 | (3.840; 74.100) | 0.0002*** |

*p<0.05; *** p<0.001; **** analysis only on 123 patients (60 UMCDK, 63 UKA)
